# 

**Published:** 1875-10

**Authors:** 


### TABLE OF CONTENTS.

ORIGINAL COMMUNICATIONS.

Differential Symptoms of Epidemic Sore Throat and Diphtheria. By A. L. Knight, M. D. .... 577

What are the Causes of the Venous Circulation? By J. G. Knox, A. B., M. D. .... 582

Accumulation and Inspissation of Cerumen. By Swan M. Burnett, Knoxville, Tenn. .... 587

Case of Retroversio n of the Uterus in the Sixth Month of Pregnancy—Forcible Reduction—Peritonitis—Recovery. By Patrick Booth, M. D., Wake county, N. C. .... 594

The Old vs. The New. By Old Fogy .... 597

SELECTIONS.

A New Antipruritic Remedy. By L. Duncan Bulkley, A. M., M. D., of New York .... 598

Adhesion of the Placenta. By J. G. Swayne, M. D .... 602

"Warm and Hot Water in Surgery." By C. Henri Leonard, M. D., Detroit, Mich .... 607

EXTRACTS AND GLEANINGS.

Ladies' Slipper as an Emmenagogue—By G. H. Batte, M. D.—611. The Necessity of Active Treatment in all Acute Diseases of the Throat—By A. G. Smythe, M. D.—612. Wet Strapping—613. Ovariotomy Complicated with Pregnancy—Cæsarean Operation—Cure—614. Perineal Tumor of Fœtus an Impediment to Delivery—615. Failure of Bromide of Iron in Chorea—616. Carcinoma—618. Removing the Ovum in Cases of Abortion—619. Oxide of Zinc in Infantile Diarrhœa—620. Therapeutic Notes—Anti-Catarrhal Pills—621. Dressing for Ulcers—621. Treatment of Lead-Colic by Chloroform—621. In Syphilis—621. Tetanus, Twenty-eight Days After Injury, Treated by Chloral—622. Poisoned by a Hat—622. Treatment of Diphtheria and Scarlet Fever—623. Hyperidrosis Cured by the Local Application of Diachylon Plaster—623. Incontinence of Urine—623. Treatment of Abscess of Breast by Compressed Sponge—624. Premature Baldness—Treatment—624. Horse-Hair for Sutures—624. The Value of Tar in Bronchial Catarrh and Winter Cough—625. Glycerin in Medical Pastes—625· Incontinence of Urine—Cure—626. How to Cure Phorrhœa Alveolaris—626. Treatment of Gonorrhœa—627. On the Use of Oxide of Zinc in the Treatment of Diarrhœa—627. Varicose Veins—627. Another "Miracle"—628. Obstinate Vomiting in Pregnancy Treated by Dilatation of the Os Uteri—628. Treatment of Erythema and Intertrigo by Means of Collodion—628. Homœopathic Medicines must not be Shaken—629. Rheumatism Treated by a Non-Nitrogenous Diet—629. Effects of the Inhalation of Chloroform, During Parturition, on the Fœtus—629. Sciatica Cured by Eating—630. Treatment of Whooping Cough—630. Ethiops Mineral—630. Liniment for Scabies—630. School for Nuses—630.

EDITORIAL AND MISCELLANEOUS.

To Our Delinquent Subscribers .... 631

Cincho-Quinine .... 631

Cincho-Quinine .... 633

London Doctors .... 635

An Error Corrected. .... 637

List of Payments .... 639

